# Assembly-Induced Emission Enhancement in Glutathione-Capped Bimetallic Gold and Copper Nanoclusters by Al^3+^ Ions and Further Application in Myricetin Determination

**DOI:** 10.3390/molecules28020758

**Published:** 2023-01-12

**Authors:** Hao-Jie Bai, De-Yan Qi, Hong-Wei Li, Yuqing Wu

**Affiliations:** 1 State Key Laboratory of Supramolecular Structure and Materials, College of Chemistry, Jilin University, 2699 Qianjin Street, Changchun 130012, China; 2 Institute of Theoretical Chemistry, College of Chemistry, Jilin University, 2 Liutiao Road, Changchun 130023, China

**Keywords:** bimetallic nanoclusters, aluminum ion, myricetin, assembly-induced emission enhancement, two-step responses

## Abstract

A significant emission enhancement (>100-fold) of glutathione-capped bimetallic gold and copper nanoclusters (AuCuNC@GSH) was achieved by assembling with Al^3+^ ions and by assembly-induced emission enhancement (AIEE). Further chelation of myricetin to Al^3+^ resulted in emission quenching of AuCuNC-Al^3+^, which was applied to specifically detect myricetin. Two linear responses were shown in the range of 0–1.5 μM and 1.5–50 μM, separately, leading to a low limit of detection at 8.7 nM. The method was successfully and accurately applied to myricetin determination in grape juice, which showed good application for real samples. Finally, the in-depth mechanism revealed that both the chelation of myricetin and Al^3+^ and the inner filter effect (IFE) between myricetin-Al^3+^ and AuCuNC-Al^3+^ greatly contributed to the quenching response of myricetin. Therefore, the present study provides an easy way to improve the fluorescence property of metal nanoclusters. Additionally, it supplies a cost-effective and easily performed approach to detect myricetin with high selectivity and sensitivity.

## 1. Introduction

Metal nanoclusters (MNCs), as a new type of fluorescent material, have attracted wide interest due to their unique properties, such as strong luminescence, low toxicity, easy preparation, large Stokes shift, and so on [1,2]. In addition, their luminescence range is tunable from the visible to the near-infrared, which make them widely used in optical sensing and bioimaging or biolabeling [3,4]. However, the current challenge in this field of MNCs is their low-fluorescence quantum yields (QYs), which greatly limits their practical applications [5,6]. Recently, the assembly/aggregation-induced emission (AIE) properties of MNCs have been reported, which largely enhanced their QYs and stability [7,8]. For example, Dutta et al. reported on AIE-activated CuNCs and applied them to cancer cell imaging, which provided a new platform to differentiate cell lines based on the intracellular AIE [9]. Rad et al. constructed a stable system by employing discoidal phospholipid bicelles to encapsulate Au_25_(SC_n_H_2n+1_)_18_ (n = 4–16), which increased the emission by 20–60 times compared with that of the free AuNCs in chloroform [10]. However, assembly/aggregation-induced emission enhancement (AIEE) is also used to describe the improvement in the luminescent properties of AuNCs with weak emission rather than no luminescence [11]. The essential mechanism is mainly attributed to the fact that the nonradiative decay is minimized because of the restricted molecular motions of the protected motifs on MNCs’ surface upon assembly/aggregation [12]. In brief, AIE/AIEE is an effective way to improve the photoluminescence property and stability of MNCs after synthesis [13,14].

Myricetin, as a flavonol compound, is widely distributed in natural plants and daily foods, including fruits, vegetables, honey, red wine, tea, and so on [15,16]. Modern pharmacological studies show that myricetin possesses multiple biological functions such as anti-inflammatory, antitumor, antibacterial, antiviral, and even antiobesity effects [17,18,19,20]. Myricetin was also used in cosmetics to regulate the ultraviolet-B-induced H_2_O_2_ generation or to inhibit the production of photoaging free radicals in skin [21]. Considering the wide distribution and benefits to human health, several traditional determination methods, such as capillary electrophoresis [22], chemiluminescence [23,24,25], high-performance liquid chromatography [26,27], spectrophotometry, and voltammetry [28,29,30], have been established for myricetin determinations. These methods have enough sensitivity and accuracy, but the complicated operations and cost instruments limit their applications. In comparison, the fluorescence method is more suitable for myricetin detection because of its rapidity, sensitivity, real-time, and in situ advantages in detection. The current challenge with myricetin, however, is the difficulty of being distinguished from other flavonoids because of their high similarity in structures [24,28]. Therefore, a highly selective and sensitive fluorescence sensor for myricetin determination needs to be developed.

In the previous work, we prepared red-emitted bimetallic gold and copper nanoclusters (AuCuNC@GSH) by employing GSH as a reductant and stabilizing agent [31]. However, the low quantum yield of emission significantly limited their application, which needed further improvement. Because Al^3+^ has the advantages of high natural abundance and cheap availability, we used it to replace Ce^3+^. Al^3+^ was introduced to construct an assembly with AuCuNC@GSH, which resulted in a larger particle of AuCuNC-Al^3+^ and an obvious emission enhancement (>100-fold). However, the quenching of myricetin on AuCuNC-Al^3+^, linearly endowed, could be further used to detect myricetin, which showed high selectivity and excellent sensitivity for myricetin in aqueous solution. Moreover, the high accuracy for the myricetin-spiked grape juice ensured the practical application of the developed method. Particularly, the in-depth mechanism investigation, both on Al^3+^-induced assembly enhancement and the myricetin-induced quenching of emission, laid the foundation to develop more efficient fluorescent approaches for the detection of myricetin and other flavonols.

## 2. Results and Discussion

### 2.1. Construction and Characterization of AuCuNC-Al^3+^ Assembly

**Figure 1:**
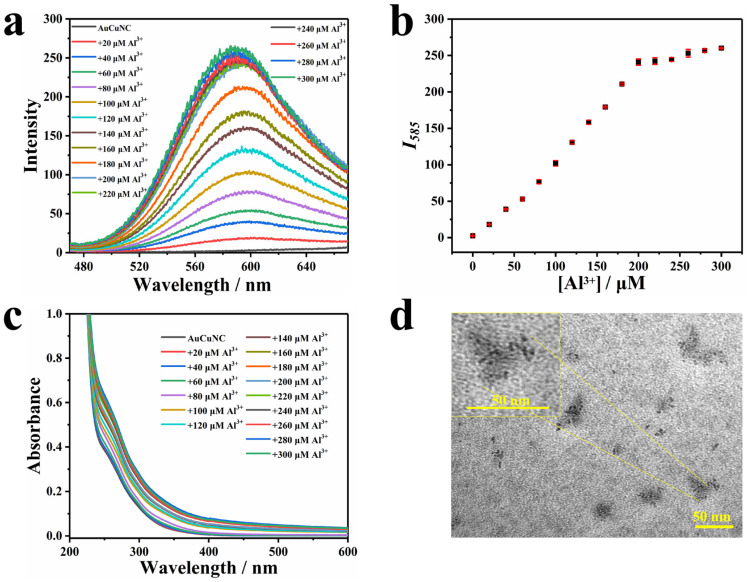
(**a**) Fluorescence and (**c**) UV–Vis absorption spectra of AuCuNC@GSH (0.050 mg∙L^−1^) in the presence of different amount of Al^3+^ (0–300 μM) in MES-NaOH buffer (pH = 6.0); (**b**) the corresponding intensity changes at 585 nm (λex = 350 nm); (**d**) typical TEM image of AuCuNC@GSH (0.050 mg∙L^−1^) in the presence of Al^3+^ (200 μM).

The procedures for the preparation and characterization of AuCuNC@GSH were the same as previously reported [31], which produced a weak emission centered at 606 nm. To achieve the cross-link of protected ligand via electrostatic interaction, the metal cations were introduced to the solution containing AuCuNC@GSH. As shown in Figure 1a, in the presence of different amounts of Al^3+^, the red emission of AuCuNC gradually increased, accompanied by a slight blue shift, finally resulting in a strong emission at 585 nm and a more than 100-fold enhancement. The fluorescence quantum yields (QYs) were also examined by using a fluorescence spectrophotometer with an integrating sphere, which showed that the QYs of AuCuNC increased from 0.3% to 10.45% in the presence of Al^3+^. It shows a good linear fluorescence response until a saturation concentration at 200 μM in the plot of Al^3+^ concentration versus corresponding fluorescence intensity (Figure 1b). Therefore, 200 μM Al^3+^ was selected as the optimal concentration for the assembly construction. Under the irradiation of UV light (365 nm), the emitting color gradually turned from pale to red with more Al^3+^ joining, although it remained almost colorless under sunlight (Figure S1 in Supplementary Materials). However, none of the other 11 metal ions, including Na^+^, K^+^, NH_4_^+^, Mg^2+^, Ca^2+^, Co^2+^, Ni^2+^, Cu^2+^, Cd^2+^, Pb^2+^, or Fe^3+^, induced emission enhancement in AuCuNC@GSH (Figure S2). Therefore, among these metal ions, only Al^3+^ displayed strong emission enhancement of AuCuNC. It is well known that in aqueous solutions, the existing form of Al^3+^ is very complex, which is highly dependent on the pH of the solution. Therefore, the pH effects of Al^3+^ on the AuCuNCs emission were also examined. When the experiment was performed in MES-NaOH buffer at pH = 6.0, Al^3+^ could induce significant fluorescence enhancement in AuCuNCs, while at pH = 6.8, it only showed a weak fluorescence enhancement (Figure S3). Such difference of fluorescence response is attributed to the existing forms of Al^3+^ at different pH conditions. In addition, the UV–Vis absorption spectra were also accordingly recorded. As shown in Figure 1c, a strong absorption peak centered at 260 nm and a broad one around 295 nm were exhibited for AuCuNC@GSH. In the presence of Al^3+^, both gradually increased, accompanied by baseline drifting. Based on previous reports [7,32], the peak around 295 nm was ascribed to the absorption band of ligand-to-metal charge transfer (LMCT), corresponding to that from the electron-rich nitrogen atom in GSH to the metal core of AuCuNC and being regarded as the origination of photoemission. No new band appeared between 350 and 500 nm, indicating that little change occurred for metal core upon Al^3+^ binding.

The emission blue shift and enhancement induced by Al^3+^ observed here were similar to the AIEE of AuNC previously reported [7,8,9,10,11,12,13,14]. As the AIEE property was essentially reflected at the surface of nanoclusters, the surface potential of AuCuNC@GSH was then monitored upon titrating with Al^3+^. As shown in Figure S4b, the zeta potential obviously increased from negative to positive values, along with more Al^3+^ joining, which confirmed the opposite surface charges between them and suggested a possible electrostatic driving force for the assembly between AuCuNC@GSH and Al^3+^. Therefore, we speculate that the strong binding of Al^3+^ to GSH at the surface of AuCuNC leads to efficient assembly through electrostatic interaction. To confirm this, dynamic light scattering (DLS) was conducted to monitor the particle size changes. As shown in Figure S4a, it gradually increased in the presence of more Al^3+^ and reached 50 nm at 200 μM Al^3+^. The results suggest that the electrostatic interaction between AuCuNC@GSH and Al^3+^ induces the assembly and forms large particles. To further testify this hypothesis, the particle size and morphology changes in AuCuNC@GSH were then monitored by using transmission electron microscopy (TEM). As shown in Figure S5, the AuCuNC@GSH were monodispersed in a very small size, and the mean diameter was calculated to be 2.20 nm (n = 300), which is consistent with previously reported results [31]. However, the dispersed nanodots merged into large spherical particles (≥30 nm) in the presence of Al^3+^ (Figure 1d), demonstrating that AuCuNC@GSH and Al^3+^ directly assembled into large particles. Taken together, the assembly process is driven by the electrostatic interaction between AuCuNC@GSH and Al^3+^. Consequently, the cross-linking between AuCuNC@GSH and Al^3+^ limits the rotation and/or vibration of the functional groups of GSH at metal surfaces, which enhances the emission through reducing the nonradiative decay. Therefore, the Al^3+^-induced fluorescence enhancement in AuCuNC@GSH is ascribed to the AIEE effect.

### 2.2. Determination of Myricetin Using AuCuNC-Al^3+^

**Figure 2:**
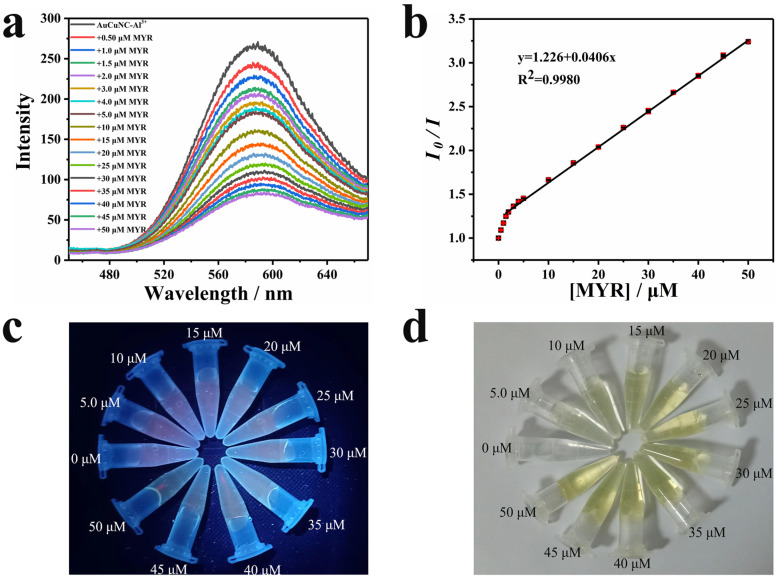
(**a**) Fluorescence spectra of AuCuNC-Al^3+^ (0.050 mg∙L^−1^, 200 μM) in the presence of different amounts of myricetin (MYR, 0, 0.50, 1.0, 1.5, 2.0, 3.0, 4.0, 5.0, 10, 15, 20, 25, 30, 35, 40, 45, 50 μM) in MES-NaOH buffer (pH = 6.0); (**b**) the corresponding intensity ratio changes at 585 nm (the initial intensity over the measured value, λ_ex_ = 350 nm), and photograph of AuCuNC-Al^3+^ (0.050 mg∙L^−1^, 200 μM) in the presence of different amounts of myricetin (0–50 μM) in MES-NaOH buffer (pH = 6.0) under (**c**) UV light (365 nm) and (**d**) sunlight.

As there was a strong chelation of flavonols to Al^3+^, the assembly of AuCuNC-Al^3+^ was applied to detect these compounds [33,34]. Interestingly, the emission gradually quenched upon titrating myricetin to the solution of AuCuNC-Al^3+^ in MES-NaOH buffer (pH = 6.0) (Figure 2a). Especially, the fluorescence quenching response is efficient and requires only mild conditions, which meets the requirements of fluorescent sensors and can be used for myricetin determination. The linear response of the emission intensities of AuCuNC-Al^3+^ at 585 nm to the myricetin concentrations in a wide range of 1.5–50 μM (Figure 2b) validated it well. The titration using diluted myricetin illustrates two linear responses in the ranged of 0–1.5 μM and 1.5–5.0 μM. A limit of determination (LOD), 8.7 nM, was achieved by calculating the three-fold standard deviation (3δ) of the blank intensity corresponding to the linear response plot (Figure S6). Therefore, the assembly of AuCuNC-Al^3+^ can be used as a fluorescence sensor to quantitatively detect trace amounts of myricetin in solution. Along with the emission becoming weaker (Figure 2c), the color of the solution gradually turned from colorless to yellow (Figure 2d). As 50 μM myricetin is colorless (Figure S7), this indicates that a new substance or structure is possibly formed in the presence of myricetin.

A comparison of the present method with previous approaches for myricetin determination is listed in Table S1. Both the LOD and linear range of the proposed method are better than or at least comparable to those of previous approaches [25,26,27,28,29,30,31]. Particularly, most previous methods require expensive instruments, professional techniques, or complicated sample preparing; in contrast, our fluorescent method needs only a low-cost spectrometer with a very simple preparation, making it more suitable for routine analysis of myricetin in real samples. Therefore, the developed sensor can be used for efficient and accurate myricetin determination.

**Figure 3:**
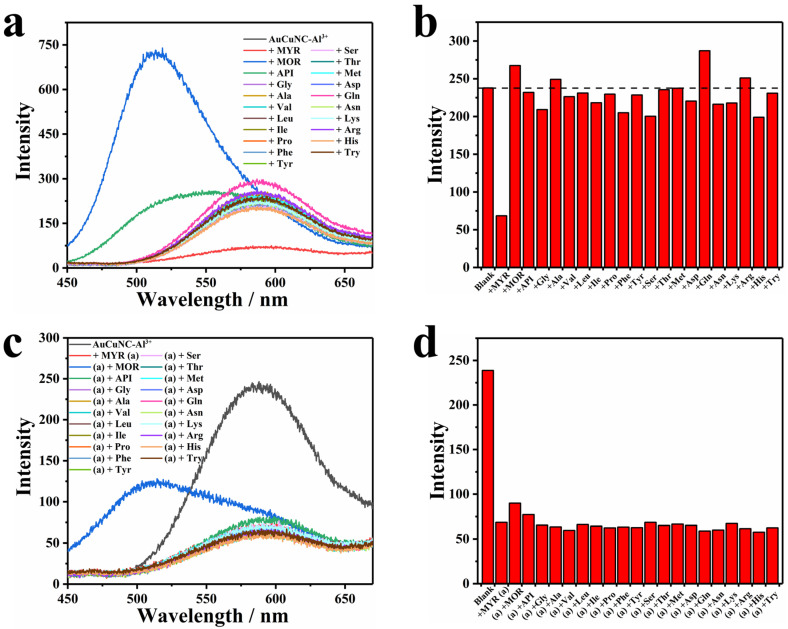
(**a**) Fluorescence spectra and (**b**) the corresponding intensity changes (585 nm) of AuCuNC-Al^3+^ (0.050 mg∙L^−1^, 200 μM) in the presence of different kinds of amino acids (50 μM) and other flavonols in MES-NaOH buffer (pH = 6.0); (**c**) fluorescence spectra; and (**d**) the corresponding intensity changes (585 nm) of AuCuNC-Al^3+^ (0.050 mg∙L^−1^, 200 μM) in the absence and presence of myricetin (50 μM), and the mixture of myricetin and equivalent amino acids or other flavonols (50 μM) in MES-NaOH buffer (pH = 6.0; myricetin, morin, apigenin, glycine, alanine, valine, leucine, isoleucine, proline, phenylalanine, tyrosine, serine, threonine, methionine, aspartic acid, glutamine, asparagine, lysine, arginine, histidine, and tryptophan are abbreviated as MYR, MOR, API, Gly, Ala, Val, Leu, Ile, Pro, Phe, Tyr, Ser, Thr, Met, Asp, Gln, Asn, Lys, Arg, His, and Try, respectively).

To verify the selectivity of the developed method to myricetin, we also applied AuCuNC-Al^3+^ to detect amino acids commonly found in the human body. There was almost no emission change that could be observed in the presence of each one, except for asparagine, which somewhat induced emission enhancement (Figure 3a). Such selectivity is more clearly shown by a bar chart of intensity variation (Figure 3b), which indicates the specificity of quenching response by myricetin. Other flavonols (morin and apigenin) with similar molecular structures as myricetin (Figure S8) were also introduced to the solution of AuCuNC-Al^3+^, one by one. As exhibited in Figure 3a, they induced another emission peak around 500 nm but maintained the luminescence of AuCuNC-Al^3+^ at 585 nm unchanged. Therefore, focusing on the luminescence intensity at 585 nm, none of them produced interference on myricetin detection (Figure 3b). That is, a high selectivity of AuCuNC-Al^3+^ toward myricetin over other interferences was achieved. In addition, in order to validate the practical application of AuCuNC-Al^3+^ as a fluorescence sensor for myricetin, competition experiments were also conducted by mixing myricetin (50.0 µM) with an equivalent competitor. As illustrated in Figure 3c,d, little interference was observed in the presence of each, suggesting that AuCuNC-Al^3+^ can be used for myricetin detection in practice. Therefore, the high selectivity and anti-interference ability of AuCuNC-Al^3+^ toward myricetin will extend its practical applications in real sample detection.

### 2.3. The Intrinsic Mechanism of Quenching Response of AuCuNC-Al^3+^ to Myricetin

As exhibited in Figure S9, the maximum absorption peak of myricetin (centered at 370 nm) overlaps with the excitation spectra of AuCuNC-Al^3+^, which suggests an inner filter effect (IFE) is possible for the quenching response toward myricetin. UV–Vis absorption spectra of AuCuNC-Al^3+^ in the buffer were measured upon adding different amounts of myricetin (Figure S10). Along with the addition of myricetin, a new absorption peak at 435 nm was generated and gradually increased, and another peak at 370 nm appeared in the presence of a large amount of myricetin (≥40 μM). As the typical absorption of myricetin is at 370 nm, the absorption peak at 435 nm could represent a new substance or structure generation. The time effect of this process was further investigated. As illustrated in Figure S11, the absorption peak at 435 nm appeared and gradually increased in the presence of myricetin, while the original absorption of myricetin at 370 nm was gradually quenched, which indicates that the new substance or structure was converted from myricetin. When time-dependent fluorescence spectra were analyzed, observations showed that the emission gradually quenched and the quenching rate gradually decreased with time, which took about 60 min to reach the final state (Figure S12), which is consistent with the absorption changes. The results indicate that the quenching response is a gradual process, which suggests that assembly reconstitution or new substance generation occurs during the process, and these were explored as follows.

When titrating Al^3+^ to the solution of myricetin, the absorption peak at 370 nm also moved to 435 nm (Figure S13), which could be attributed to the chelation with Al^3+^ and formation of myricetin-Al^3+^ complex [35]. Consequently, the new absorption peak at 435 nm was attributed to the strong chelation between myricetin and Al^3+^. The results prove that the addition of myricetin destroys, or at least changes, the previous assembly of AuCuNC-Al^3+^, which consequently quenches the emission. Though the characteristic absorption shifts from 370 to 435 nm, compared with the excitation spectra of AuCuNC-Al^3+^, it still possesses some overlapped region, which indicates that the IFE is still an important factor leading to the fluorescence quenching. To test it, we titrated the complex of myricetin-Al^3+^ (1:1) into the system to exclude the effect of competitive chelation of Al^3+^ on the assembly structure of AuCuNC-Al^3+^. As shown in Figure S14, the complex of myricetin-Al^3+^ gradually quenched the emission of AuCuNC-Al^3+^, which demonstrates that IFE is an important fluorescence quenching factor. Compared with the previous reports, the quenching response of ATP to AuCuNCs-Ce^3+^ was attributed to the strong interaction between Ce^3+^ and ATP, which induced disassembly of AuCuNCs-Ce^3+^ and recovery of the weak AuCuNC@GSH emission [31]. In the present study, the quenching response is attributed to the IFE between myricetin-Al^3+^ and AuCuNC-Al^3+^ through the chelation between myricetin and Al^3+^.

**Figure 4:**
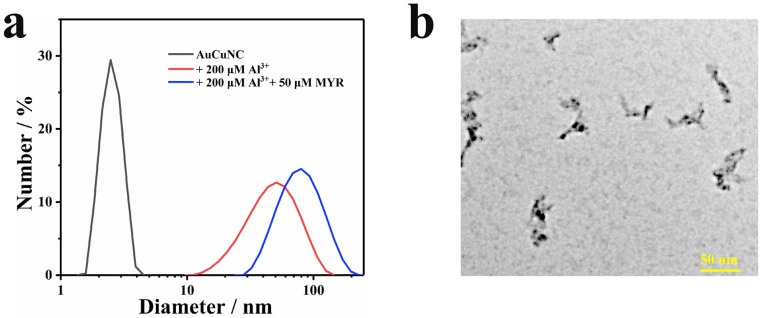
(**a**) Dynamic light scattering (DLS) of AuNCs@GSH (0.050 mg∙L^−1^) in the absence and presence of Al^3+^ (200 μM) and myricetin (50 μM); (**b**) typical TEM image of AuNCs-Al^3+^ (0.050 mg∙L^−1^, 200 μM) in the presence of myricetin (MYR, 50 μM).

**Scheme 1:**
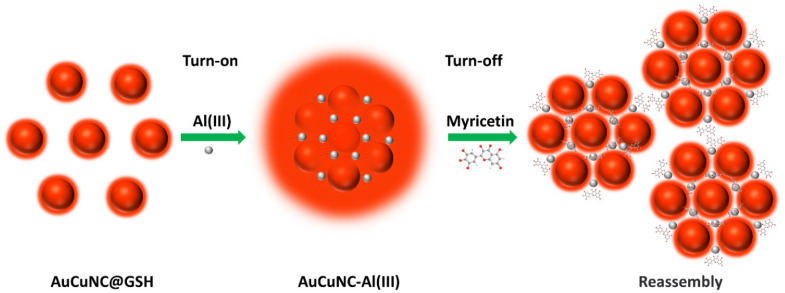
Schematic illustration of the assembly process of AuCuNCs@GSH and Al^3+^, and further application in myricetin determination.

To further examine the effect of myricetin addition on the structure of AuCuNC-Al^3+^ assembly, DLS and TEM were performed. As shown by DLS, the hydrated particle size of the assembly increased from 50 nm to 70 nm (Figure 4a). The addition of myricetin did not destroy the particle size of the assembly, beyond increasing it to a certain extent, indicating that myricetin also participates in the internal structure of the assembly and induces a multivariate complex. The typical TEM image shows a clear cross-linking structure of the assembly (Figure 4b), highly in line with that of DLS. Therefore, the assembly reconstitution can be ascribed to the chelation between myricetin and Al^3+^, which induces the structure changes of assembly; additionally, the hydrophobic interactions between myricetin inducing the cross-linking (Scheme 1). The results demonstrate that the fluorescence quenching can be attributed to the IFE between myricetin-Al^3+^ and AuCuNC-Al^3+^ and chelation between myricetin and Al^3+^, which induces structure changes in the assembly.

### 2.4. The Detection of Myricetin in Grape Juice

To verify the application of the proposed method in practice, the AuCuNC-Al^3+^ assembly was further applied for myricetin determination in grapes. The fresh grapes were pressed, filtered, and centrifuged to grape juice. Then, the gradual addition of different volumes of juice to the solution containing AuCuNC-Al^3+^ assembly induced obvious linear fluorescence quenching up to 9.0 μL (Figure S15). Finally, the myricetin concentrations in grape juice were calculated to be 181.4 μM, highly in line with that obtained by the HPLC analysis (Table S2). Therefore, the amount of myricetin in grape juice was evaluated to be 0.0508 g∙L^−1^, corresponding to about 29 mg myricetin per kilogram of freshly harvested grapes. In addition, the determination of myricetin was also carried out by spiking a series of fixed amounts of myricetin into the collected grape juice. Based on the developed fluorescence method, the recovery rates of myricetin were calculated to be between 97.72 and 102.02%, accurate enough to fit the needs of the practical applications. Therefore, this further confirmed that the constructed AuCuNC-Al^3+^ assembly can be used to determine myricetin in real food samples.

## 3. Experimental Method

### 3.1. Materials and Reagents

Myricetin, morin, and apigenin were purchased from TCI Development Co., Ltd. (Shanghai, China); 4-morpholineethanesulfonic acid (MES, >99.9%), glutathione, and all amino acids (AAs, >99.9%) were purchased from Aladdin Reagent. HAuCl_4_·3H_2_O, AlCl_3_·6H_2_O, and CuSO_4_ were purchased from Beijing Chemical Factory (Beijing, China) and the purities of these were higher than 99%. All these chemicals were obtained from commercial suppliers and used without further purification. Distilled water (ρ = 18.2 MΩ·cm, 25 °C) was obtained from a water purification system (Millipore Milli-Q), while the 10 mM MES-NaOH buffer (pH = 6.0) was prepared with 10 mM MES and NaOH in such water.

### 3.2. Instruments and Sample Preparations

Fluorescence spectra were recorded on a Shimadzu (Kyoto, Japan) RF-5301PC spectrophotometer. All fluorescence measurements of AuCuNC@GSH, the assembly of AuCuNC-Al^3+^, and the responses to myricetin, as well as its interferences, were performed in 10.0 mM MES-NaOH buffer (pH = 6.0) with a fixed excitation wavelength at 350 nm. UV–Vis absorption spectra were recorded on a Shimadzu (Kyoto, Japan) UV-3600 spectrophotometer, performed in a 1 cm × 1 cm quartz cuvette (4 mL volume). To reduce the fluctuation in the measurement, the lamp was kept on for 0.5 h before use. The concentration of AuCuNC@GSH was fixed at 0.050 mg∙L^−1^ in all the testing measurements. Transmission electron microscopy (TEM) was conducted by using JEM-2200FS (Jeol Ltd., Tokyo, Japan) at an accelerating voltage of 200 kV. The sample was dripped on a copper net and dried naturally. In addition, the zeta potential and dynamic light scattering (DLS) measurements were performed with a Zetasizer Nano ZS90 (Malvern Instruments, Malvern, UK), and the data are demonstrated by numbers.

### 3.3. Preparation of AuCuNC@GSH

The synthesis process of AuCuNC@GSH was the same as that previously reported [31]. The procedure can be summarized as follows: under stirring, 5.0 mL of GSH solution (50 mg∙mL^−1^) was gradually added to 5.0 mL of CuSO_4_ solution (10 mM), and then 1.0 M NaOH solution was titrated into the system until the precipitation disappeared. After further stirring for 2 h at room temperature, 1.0 mL of HAuCl_4_ (10 mM) was added following another 3 h stirring. Finally, a light-yellow solution was obtained and stored at 4 °C for further use.

### 3.4. Detection of Myricetin

A 10 mM MES-NaOH buffer (pH = 6.0) was used as a medium for the detection of myricetin. The experiments were performed by mixing 1.0 mL of AuCuNC-Al^3+^ (0.050 mg∙L^−1^, 200 μM) in 10 mM MES-NaOH buffer (pH = 6.0) with different volumes of myricetin stock solution or grape juice. After incubating at room temperature for 60 min, fluorescence spectra were recorded under a fixed excitation wavelength of 350 nm. The relative intensity ratio (*I_0_*/*I*) of fluorescence intensity of the assembly with (*I*) and without (*I_0_*) myricetin was calculated as an analytical signal. The selectivity and competition experiments were carried out by monitoring the fluorescence spectra of other competitive substances following the same procedure. The fresh grapes were directly pressed into juice, and the suspension was first filtered through filter paper. After centrifugation and filtration, again using a PES membrane (with a pore size of 0.22 µm, from Jin Teng Ltd., Tian Jin, China), the grape juice was obtained.

## 4. Conclusions

In conclusion, Al^3+^ was initially employed to improve the emission of AuCuNC@GSH via the assembly formation of AuCuNC-Al^3+^ and the involved AIEE effects, where the interaction between metal ions and the ligand GSH restricted the intramolecular rotation and led to conversion of the energy into photons. Moreover, myricetin sensitively quenched the emission of AuCuNC-Al^3+^ in a two-step linear response, in the range of 0–1.5 μM and 1.5–50 μM, resulting in a very low limit of detection at 8.7 nM. The in-depth mechanism investigation uncovered such a quenching response was attributed to the chelation between myricetin and Al^3+^, and the IFE between myricetin-Al^3+^ and AuCuNC-Al^3+^. Finally, the proposed method was used to accurately determine myricetin in grape juice, showing high potential in practical applications. Therefore, the present study provides an assembly method to improve the fluorescence property of metal nanoclusters, supplying a highly selective and sensitive fluorescence sensor of myricetin determination.

## Data Availability

All data concerning this study are contained in the present manuscript or in previous articles, and references have been provided.

## Supplementary Materials

The following supporting information can be downloaded at: https://www.mdpi.com/article/10.3390/molecules28020758/s1, Figure S1. The photograph of AuCuNC@GSH (0.050 mg∙L^−1^) in the presence of different amounts of Al^3+^ (0–300 μM) in MES-NaOH buffer (pH = 6.0) under (a) UV light (365 nm) and (b) sunlight; Figure S2. (a) Fluorescence spectra of AuCuNC@GSH (0.050 mg∙L^−1^) in the absence and presence of different metal ions (200 μM) in MES-NaOH buffer (pH = 6.0); (b) the related emission changes at 585 nm (λex = 350 nm); Figure S3. The emission intensity changes in AuCuNC@GSH (0.050 mg∙L^−1^) to Al^3+^ (200 μM) at different pH (5.0, 5.5, 6.0, 6.5, 6.8 and 7.0); Figure S4. (a) Dynamic light scattering (DLS) and (b) zeta potential changes of AuCuNC@GSH (0.050 mg∙L^−1^) in the presence of different amounts of Al^3+^ (0–200 μM) in MES-NaOH buffer (pH = 6.0); Figure S5. (a) Typical TEM image and (b) particle size distribution statistics of AuCuNC@GSH; Figure S6. (a) Fluorescence spectra of AuCuNC-Al^3+^ (0.050 mg∙L^−1^, 200 μM) in the absence and presence of different amounts of myricetin (MYR) in MES-NaOH buffer (pH = 6.0); (b) the corresponding intensity ratio changes at 585 nm (the initial intensity over the measured value, λ_ex_ = 350 nm); Figure S7. Photograph of myricetin in MES-NaOH buffer (pH = 6.0) under sunlight; Figure S8. Chemical structures of myricetin, morin, and apigenin; Figure S9. Fluorescence excitation (black) and emission (red) spectra of AuCuNCs-Al^3+^, and the UV–vis absorption (blue) spectrum of myricetin (MYR) in MES-NaOH buffer (pH = 6.0); Figure S10. UV–vis absorption spectra of AuCuNC-Al^3+^ (0.050 mg∙L^−1^, 200 μM) in the presence of different amounts of myricetin (MYR; 0–50 μM); Figure S11. Time-dependent UV–vis absorption spectra of AuCuNC-Al^3+^ (0.050 mg∙L^−1^, 200 μM) in the presence of myricetin (MYR, 20 μM); Figure S12. (a) Time-dependent fluorescence spectra and (b) corresponding intensity changes in AuCuNC-Al^3+^ (0.050 mg∙L^−1^, 200 μM) in the presence of myricetin (MYR, 20 μM); Figure S13. UV–vis absorption spectra of myricetin (50 μM) in the presence of different amounts of Al^3+^ (0–50 μM); Figure S14. Fluorescence spectra of AuCuNC-Al^3+^ (0.050 mg∙L^−1^, 200 μM) in the presence of myricetin-Al^3+^ (1:1; 0–100 μM); Figure S15. (a) Fluorescence spectra and (b) the emission intensity changes of AuCuNC-Al^3+^ in the presence of different volumes of grape juice (0–9 μL); Table S1. Comparison of the analytical performance of different methodologies toward myricetin determination; Table S2. The parameters of the present method and standard method (HPLC) for myricetin determination in grape samples.

## Sample Availability

Samples of the compounds are available from the authors.
