# Supplementary material for: Assembly-Induced Emission Enhancement in Glutathione-Capped Bimetallic Gold and Copper Nanoclusters by Al3+ Ions and Further Application in Myricetin Determination
Source: Molecules. 2023 Jan 12;28(2):758. doi: 10.3390/molecules28020758 (PMC9864343; doi:10.3390/molecules28020758)
Supplement: Supplementary file 1 [file molecules-28-00758-s001.zip › molecules-2095958-supplementary.pdf]

Assembly-induced Emission Enhancement of Glutathione Capped Bimetallic Gold and Copper Nanoclusters by Al^{3+} Ions and Further Application in Myricetin Determination

Hao-Jie Bai [1,2], De-Yan Qi [1,2], Hong-Wei Li [1,2] and Yuqing Wu [1,2,*]

[1] State Key Laboratory of Supramolecular Structure and Materials, College of Chemistry, Jilin University, 2699 Qianjin Street, Changchun 130012, P. R. China

[2] Institute of Theoretical Chemistry, College of Chemistry, Jilin University, 2 Liutiao Road, Changchun 130023, P. R. China

* Correspondence: yqwu@jlu.edu.cn

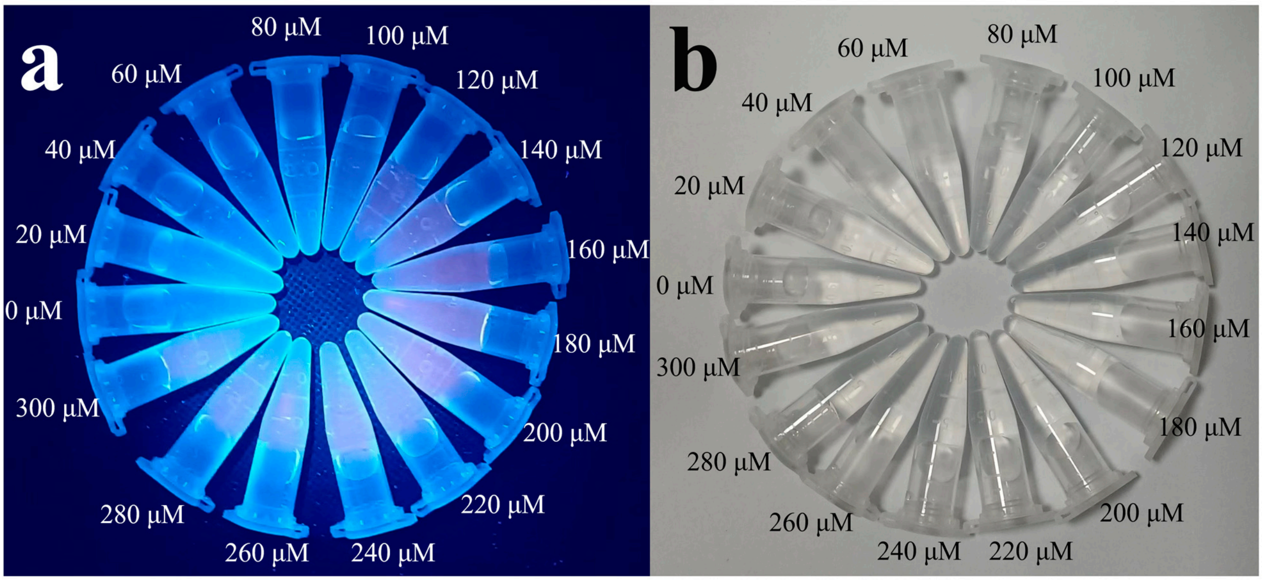

Figure S1. The photograph of AuCuNC@GSH (0.050 mg·L^{-1}) in the presence of different amounts of Al^{3+} (0 − 300 µM) in MES-NaOH buffer (pH = 6.0) under (a) UV light (365 nm) and (b) sunlight.

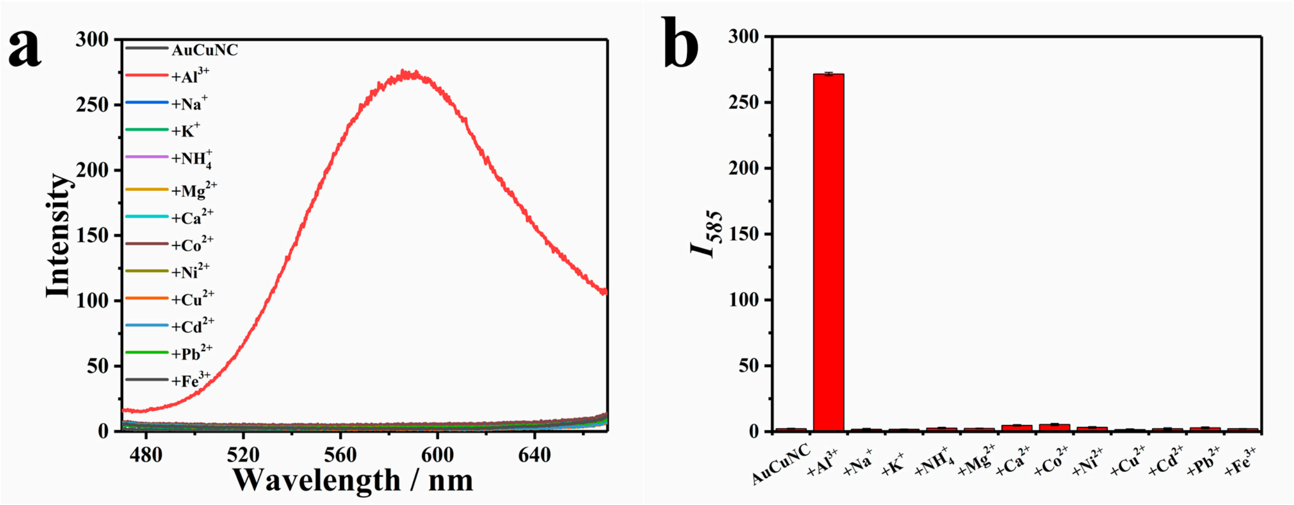

Figure S2. (a) Fluorescence spectra of AuCuNC@GSH (0.050 mg·L^{-1}) in the absence and presence of different metal ions (200 μM) in MES-NaOH buffer (pH = 6.0); (b) the related emission changes at 585 nm (λ_{ex} = 350 nm).

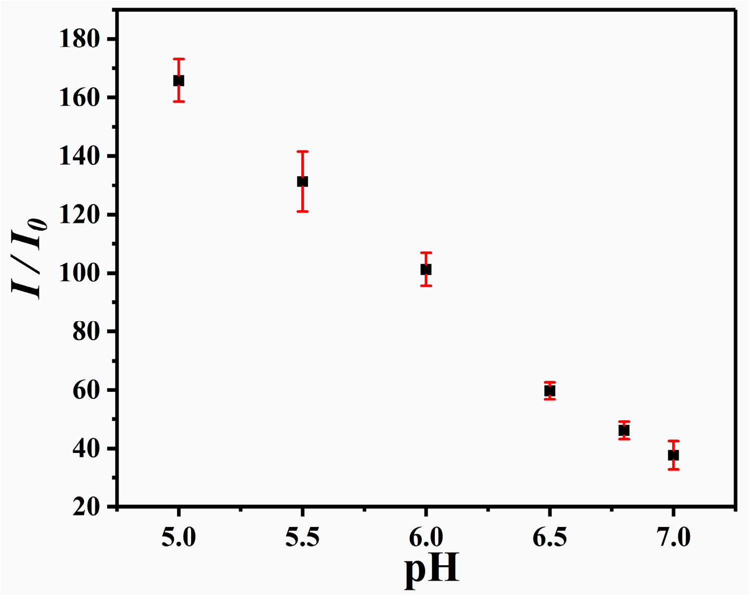

Figure S3. The emission intensity changes of AuCuNC@GSH (0.050 mg·L^{-1}) to Al^{3+} (200 μM) at different pH (5.0, 5.5, 6.0, 6.5, 6.8 and 7.0), respectively.

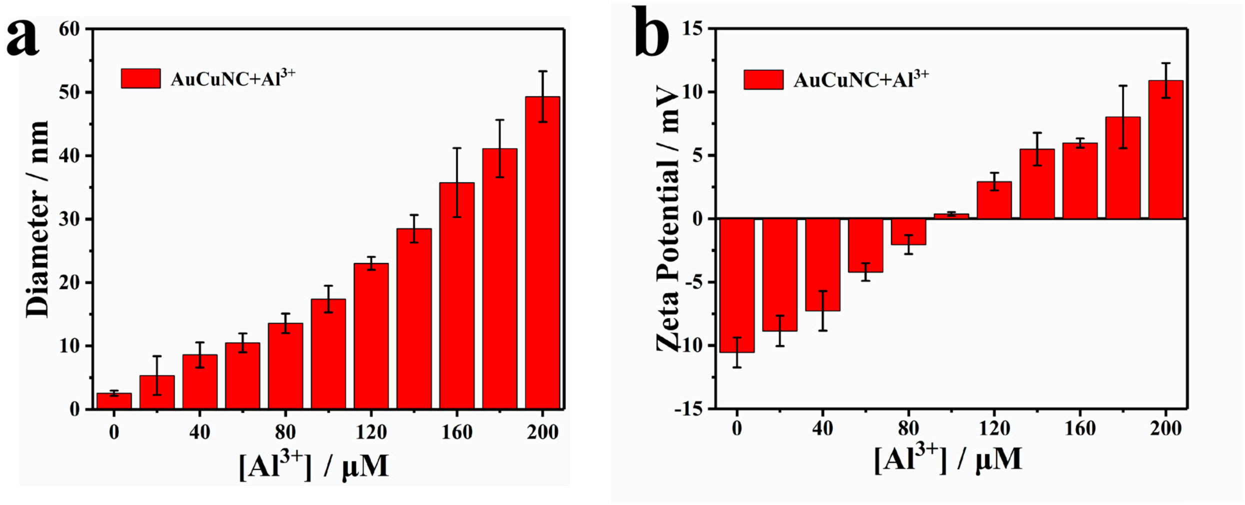

Figure S4. (a) Dynamic light scattering (DLS) and (b) Zeta-potential changes of AuCuNC@GSH (0.050 mg·L^{-1}) in the presence of different amounts of Al^{3+} (0 – 200 μM) in MES-NaOH buffer (pH = 6.0).

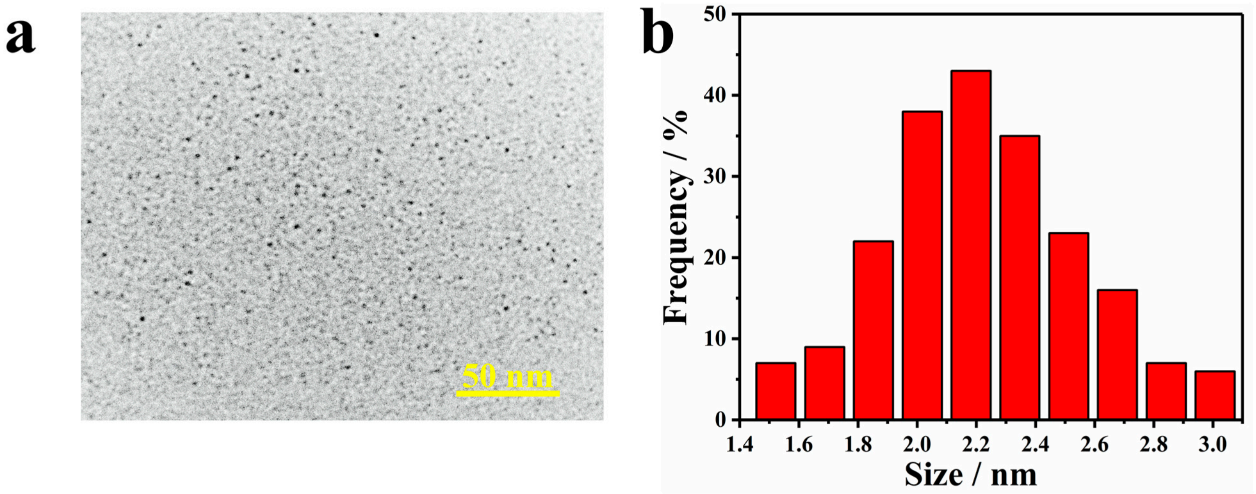

Figure S5. (a) Typical TEM image and (b) particle size distribution statistics of AuCuNC@GSH.

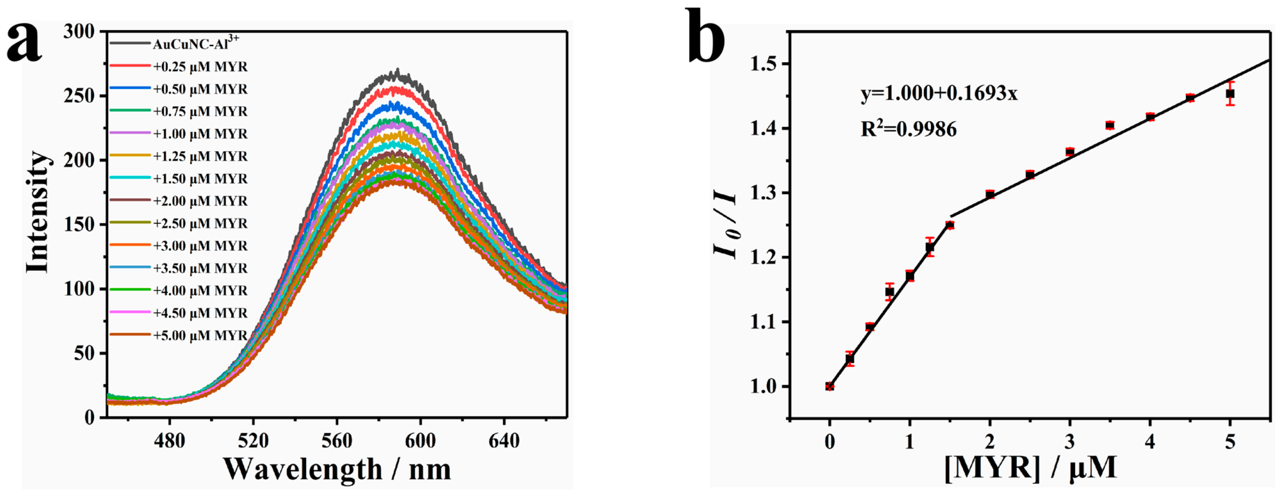

Figure S6. (a) Fluorescence spectra of AuCuNC-Al^{3+} (0.050 mg·L^{-1}, 200 µM) in the absence and presence of different amounts of myricetin (MYR) in MES-NaOH buffer (pH = 6.0); (b) the corresponding intensity ratio changes at 585 nm (the initial intensity over the measured value, λ_{ex} = 350 nm).

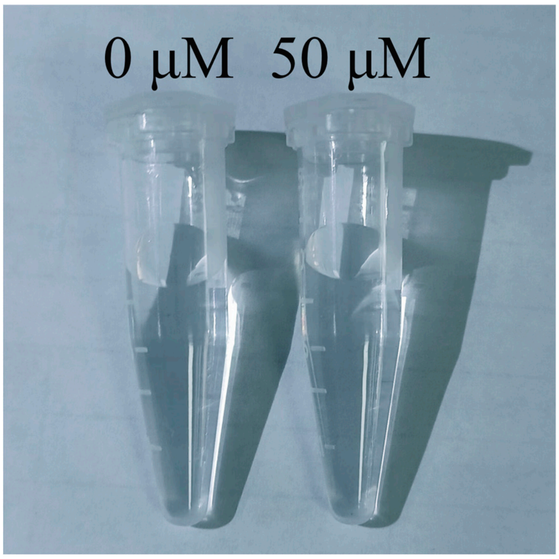

Figure S7. The photograph of myricetin in MES-NaOH buffer (pH = 6.0) under sunlight.

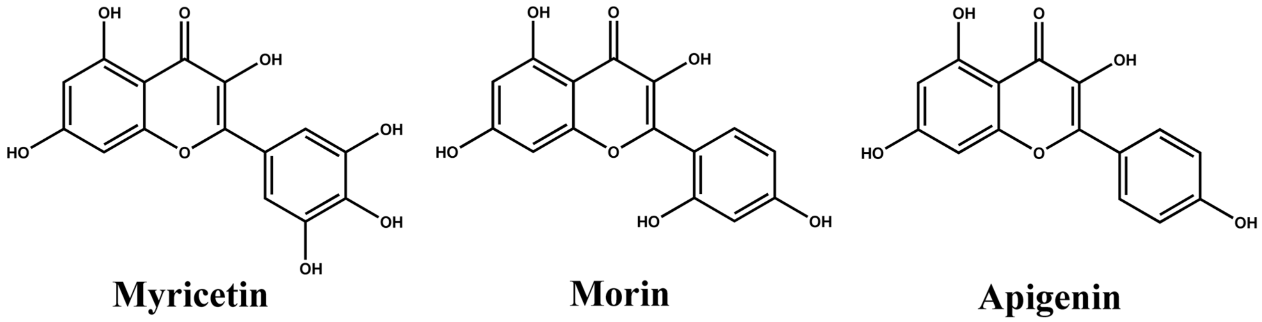

Figure S8. Chemical structures of myricetin, morin and apigenin, respectively.

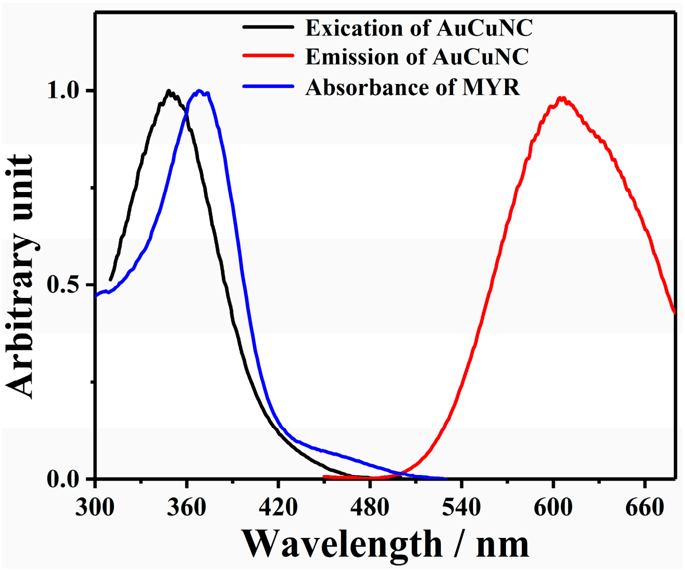

Figure S9. Fluorescence excitation (black) and emission (red) spectra of AuCuNCs-Al^{3+}, and the UV-Vis absorption (blue) spectrum of myricetin (MYR) in MES-NaOH buffer (pH = 6.0).

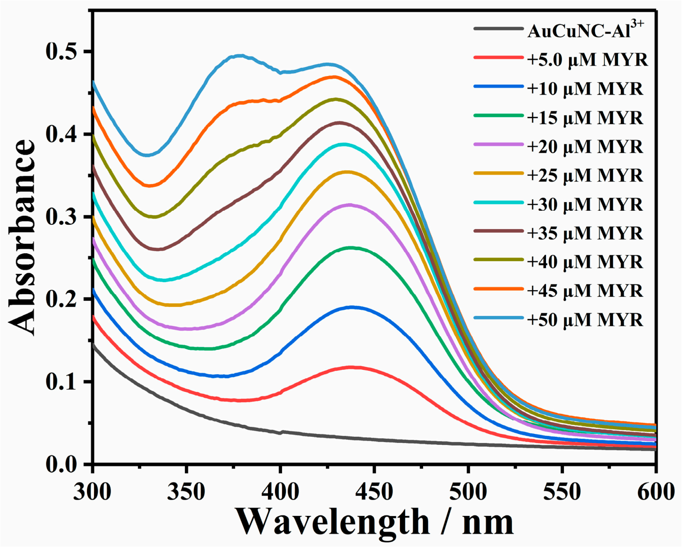

Figure S10. UV-Vis absorption spectra of AuCuNC-Al^{3+} (0.050 mg·L^{-1}, 200 µM) in the presence of different amounts of myricetin (MYR; 0 − 50 µM).

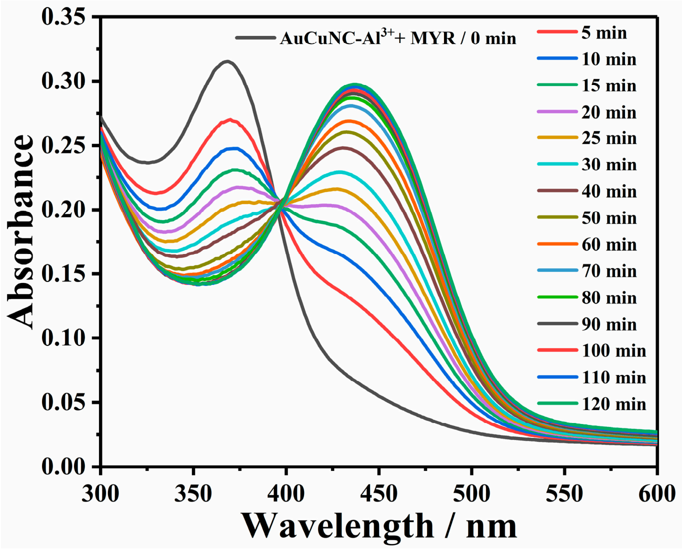

Figure S11. Time-dependent UV-Vis absorption spectra of AuCuNC-Al^{3+} (0.050 mg·L^{-1}, 200 µM) in the presence of myricetin (MYR, 20 µM).

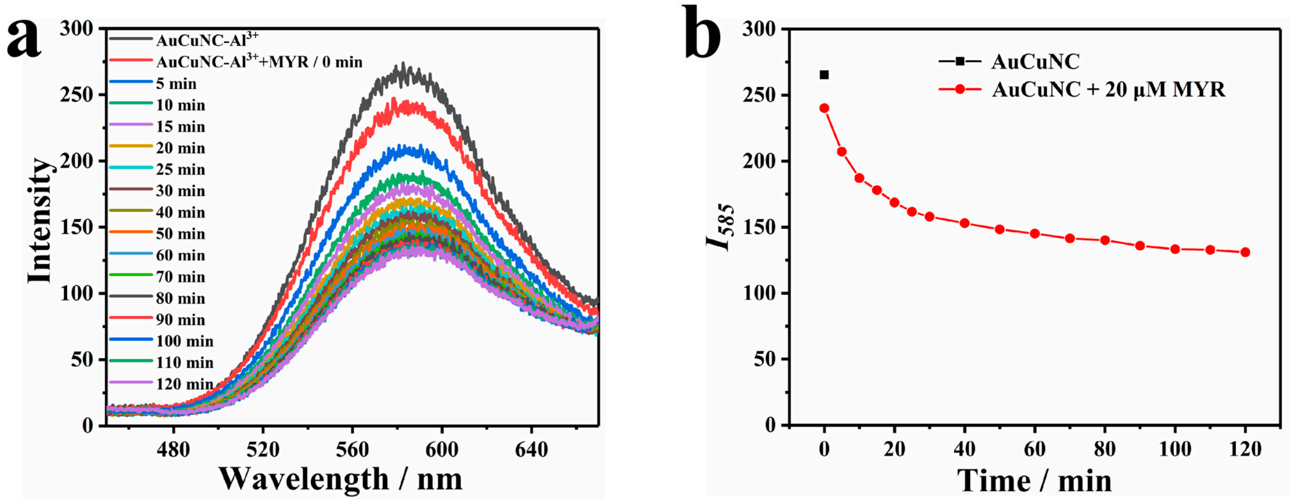

Figure S12. (a) Time-dependent fluorescence spectra and (b) corresponding intensity changes of AuCuNC-Al^{3+} (0.050 mg·L^{-1}, 200 μM) in the presence of myricetin (MYR, 20 μM).

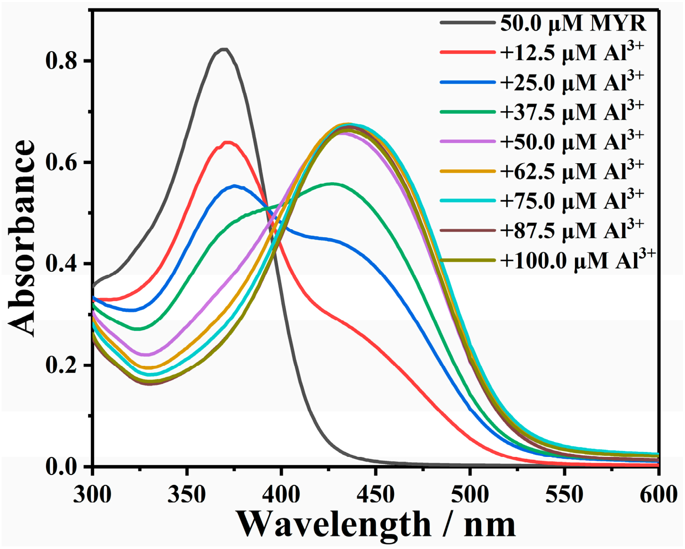

Figure S13. UV-Vis absorption spectra of myricetin (50 μM) in the presence of different amounts of Al^{3+} (0 − 50 μM).

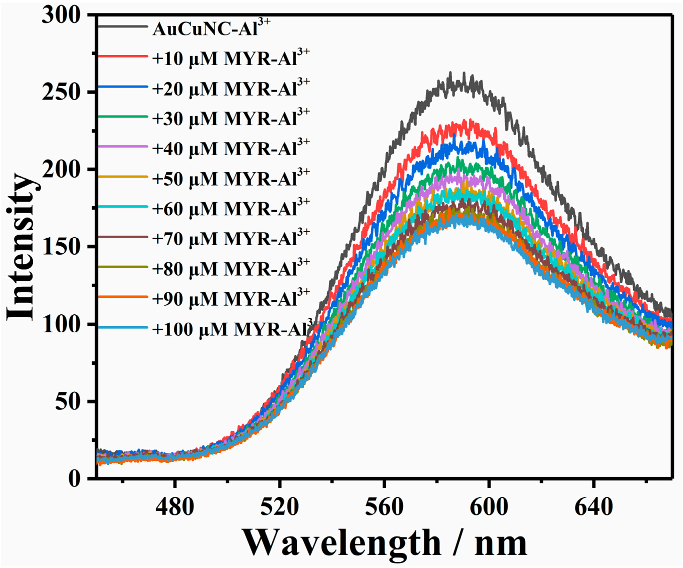

Figure S14. Fluorescence spectra of AuCuNC-Al^{3+} (0.050 mg·L^{-1}, 200 μM) in the presence of myricetin-Al^{3+} (1:1; 0 − 100 μM).

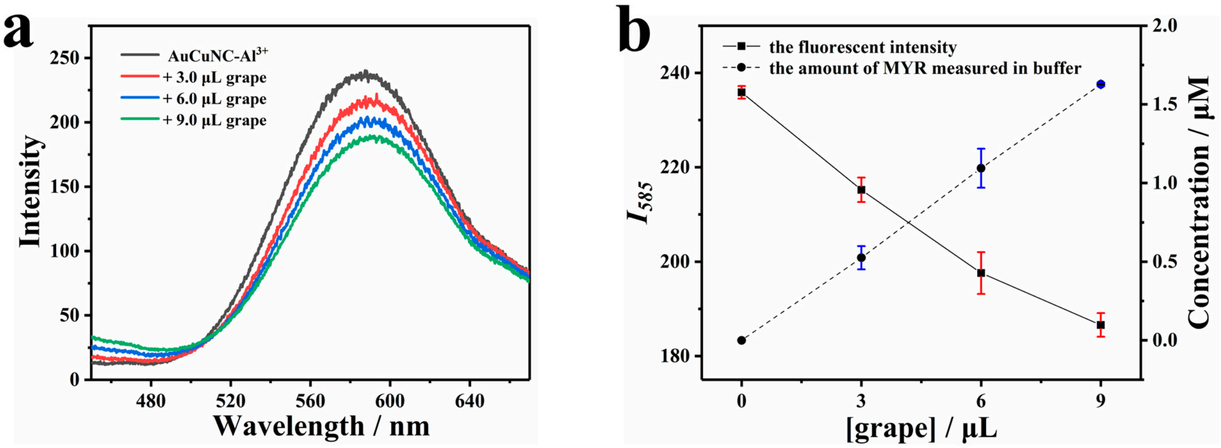

Figure S15. (a) Fluorescence spectra and (b) the emission intensity changes of AuCuNC-Al^{3+} in the presence of different volume of grape juice (0 − 9 μL).

Table S1. Comparison of the analytical performance of different methodologies towards myricetin determination.

Materials	Methodology	LOD	Linear range	Ref.
Carbon quantum dots	fluorescence	18.4 nM	1–80 μM	S1
Imprinted APBA-functionalized CdTe QDs	fluorescence	0.08 μM	0.30–40 μM	S2
Walnut septum sample	HPLC-DAD	0.24 μg/g	1–10 μg/g	S3
Ternary nanocomposite	electrochemical	3.0 nM	0.01–15 μM	S4
Ternary nanocomposite film	electrochemical	67 nM	1–110 μM	S5
Ternary nanocomposite	electrochemical	0.01 μM	0.050–50 μM	S6
AuCuNC-Al^{3+}	fluorescence	8.7 nM	1.5–50 μM	Present work

Table S2. The parameters of the present method and standard method (HPLC) for myricetin determination in grape samples.

Sample	HPLC (mM)	AuCuNC-Al^{3+} (mM)	Added (mM)	Found (mM)	Recovery (%)	RSD (%) n = 3
grape	0.180	0.181	0.50	0.625	98.48	3.631
			1.0	1.153	102.02	6.232
			2.0	2.087	97.72	3.673

Reference

S1 Liu, L.; Mi, Z.; Guo, Z.; Wang, J.; Feng, F. A label-free fluorescent sensor based on carbon quantum dots with enhanced sensitive for the determination of myricetin in real samples. *Microchem. J.* **2020**, *157*, 104956.

S2 Zhang, Y.; Tian, X.; Zhang, Z.; Tang, N.; Ding, Y.; Wang, Y.; Li, D. Boronate affinity-based template-immobilization surface imprinted quantum dots as fluorescent nanosensors for selective and sensitive detection of myricetin. *Spectrochim. Acta A.* **2022**, *272*, 121023.

S3 Kalogiouri, N. P.; Samanidou, V. F. A Validated Ultrasound-Assisted Extraction Coupled with SPE-HPLC-DAD for the Determination of Flavonoids in By-Products of Plant Origin: An Application Study for the Valorization of the Walnut Septum Membrane. *Molecule.* **2021**, *26*, 6418.

S4 Liu, C.; Huang, J.; Wang, L. Electrochemical synthesis of a nanocomposite consisting of carboxy-modified multi-walled carbon nanotubes, polythionine and platinum nanoparticles for simultaneous voltammetric determination of myricetin and rutin. *Microchim. Acta* **2018**, *185*, 414.

S5 Xing, R.; Tong, L.; Zhao, X.; Liu, H.; Ma, P.; Zhao, J.; Liu, X.; Liu, S. Rapid and sensitive electrochemical detection of myricetin based on polyoxometalates/SnO2/gold nanoparticles ternary nanocomposite film electrode. *Sensor. Actuat. B-Chem.* **2019**, *283*, 35−41.

S6 Tursynbolat, S.; Bakytkarim, Y.; Huang, J.; Wang, L. Highly sensitive simultaneous electrochemical determination of myricetin and rutin via solid phase extraction on a ternary Pt@r-GO@MWCNTs nanocomposite. *J. Pharm. Anal.* **2019**, *9*, 358−366.
